# Editorial: Primary Adrenal Insufficiency - Quality of Life and Long-Term Outcome With Current Treatment Strategies

**DOI:** 10.3389/fendo.2022.886762

**Published:** 2022-03-31

**Authors:** Daniela Esposito, Alberto Falorni, Gudmundur Johannsson

**Affiliations:** ^1^ Department of Internal Medicine and Clinical Nutrition, Institute of Medicine, Sahlgrenska Academy, University of Gothenburg, Gothenburg, Sweden; ^2^ Department of Endocrinology, Sahlgrenska University Hospital, Gothenburg, Sweden; ^3^ Section of Internal Medicine and Endocrine and Metabolic Sciences, Department of Medicine and Surgery, University of Perugia, Perugia, Italy

**Keywords:** adrenal insufficiency, quality of life, outcome, cognitive function, glucocorticoids

Primary adrenal insufficiency (PAI) is a life-threating disease characterized by inadequate secretion of glucocorticoids (GCs), mineralocorticoids (MCs), and androgens from the adrenal cortex. Conventional treatment consists of lifelong replacement with GCs (i.e. hydrocortisone or cortisone acetate, administered twice or thrice daily) and MCs (i.e. fludrocortisone 0.05 to 0.2 mg). Androgen replacement is less frequent administered in women (1).

Current replacement treatment strategies fail to restore physiological cortisol exposure and patients with PAI still have poor outcome, with increased morbidity, mortality, and impaired quality of life (QoL) (1–3). The poor outcome has mainly been related to increased exposure to cortisol and inadequate cortisol coverage during stress-related events and illness. Indeed, conventional GC replacement results in peaks and trough of GC concentrations throughout the day. Overexposure to cortisol has been associate to poor QoL, similar to that found in patients with Cushing´s syndrome (4). Similarly, underexposure to cortisol may result in fatigue, higher frequency of adrenal crisis and lower QoL (5). A novel GC replacement treatment, dual-release oral hydrocortisone has been shown to provide a more circadian-based serum cortisol profile and has be associated to a better QoL as compared to conventional therapy (6).

It is important to take in mind that MC deficiency may also play a role on long-term outcome in patients with PAI. Available evidence suggests that the current standard MC replacement may be inadequate in some patients. Indeed, signs and symptoms related to MC underreplacement are commonly reported, as hyponatremia, salt craving, and postural hypotension (7, 8). MC receptor plays an important role in cognition, thus MC underreplacement may contribute to poor QoL (9).

There are still several aspects on outcome in patients with PAI that remain poorly understood. The aim of the present Research Topic is to provide an overview of the current knowledge and a better understanding of QoL and long-term outcomes in PAI.

In one of the world’s largest registries, including 494 patients with PAI from Norway, Didriksen et al. have analysed how clinical characteristics and PAI-associated diseases affect QoL. QoL was measured using RAND-36 (generic) and AddiQoL (-30 and -8, disease-specific). QoL was significantly reduced in patients with PAI. Interestingly, men had lower scores for social functioning while in women the lowest scores were recorded for physical role. Age, gender, etiology, and comorbidity were found to be important determinants of QoL. Specifically, higher age, female sex, nonautoimmune etiology, and autoimmune comorbidities were associated with lower QoL-scores. No significant differences in QoL were recorded between different dosing regimens of GC- and MC-replacement.

Disruptions in sleep and cognitive function may affect QoL in PAI. Henry et al. have performed a comprehensive review on the current knowledge on this aspect, showing that reduced quality of sleep and cognitive impairment are common in PAI patients. Specifically, impairment in the domain of declarative memory (verbal and visual memory), but also executive functioning (attention and processing speed) are frequently observed in PAI. It is known that healthy sleep plays an important role for memory consolidation. Alterations in cortisol rhythm seem to lead to sleep disturbances and might obstruct the beneficial effects of sleep on memory consolidation. Therefore, the failure of current replacement therapy to restore physiological circadian rhythm of cortisol seems to be the main determinant of sleep disruption and cognitive impairment in patients with PAI.

Claessen et al. reviewed different clinical aspects of adrenal crisis from both the doctor’s and patient’s perspective, highlighting that there are still several challenges in the care of AI patients, as the lack of reliable biomarkers to guide GC replacement and the absence of a universally used criteria to define adrenal crisis, leading to diagnostic delay of this life-threatening condition. Moreover, the authors report the findings from a previous study (10), showing that most patients (>60%) are dissatisfied with the information they receive about the side-effects of GC replacement therapy, leading to poor adherence to therapy. Interestingly, most patients report a degree of nonadherence to GC replacement, with 36% of patients taking the GC dose later in the day than prescribed, 35% taking the GC dose at a different time of the day than prescribed, and 28% forgetting to take the GC dose. This highlights the central role of the patient in the prevention of adrenal crisis and in long-term outcome.

Hasenmajer et al. have provided an overview on the current knowledge on adrenal and extra-adrenal effects of ACTH. PAI is characterized by increased levels of ACTH which stimulate melanocytes in the skin, resulting in typical skin hyperpigmentation. However, apart from the skin effects, little is known on the systemic effects of increased levels of ACTH in PAI patients. *In vitro* studies have shown that ACTH can stimulate lipolysis (11). In animal studies, ACTH has been shown to induce insulin resistance and increase levels of pro-inflammatory adipokine (12). Whether ACTH excess may have a role in general outcomes and in metabolic complications in patients with PAI is unknown and future studies on this issue could provide useful insights to develop new therapeutic tools.

We would like to highlight and recommend the papers by Claessen et al., Hasenmajer et al., Henry et al., and Didriksen et al., as they demonstrate and review the burden of illness related to primary adrenal insufficiency. They also acknowledge the need of further refinement of therapy despite the more recent introduction of more modern oral modified release formulation for GC replacement therapy.

## Author Contributions

DE had the primary responsibility for writing the paper. AF and GJ reviewed and revised subsequent versions of the manuscript. All authors contributed to the article and approved the submitted version.

## Funding

This article was funded by the Swedish government under the ALF agreement.

## Conflict of Interest

The authors declare that the research was conducted in the absence of any commercial or financial relationships that could be construed as a potential conflict of interest.

## Publisher’s Note

All claims expressed in this article are solely those of the authors and do not necessarily represent those of their affiliated organizations, or those of the publisher, the editors and the reviewers. Any product that may be evaluated in this article, or claim that may be made by its manufacturer, is not guaranteed or endorsed by the publisher.
